# Are the Water Quality Improvement Measures of China’s South-to-North Water Diversion Project Effective? A Case Study of Xuzhou Section in the East Route

**DOI:** 10.3390/ijerph17176388

**Published:** 2020-09-02

**Authors:** Ye Pan, Yuan Yuan, Ting Sun, Yuxin Wang, Yujing Xie, Zhengqiu Fan

**Affiliations:** Department of Environment Science and Engineering, Fudan University, Shanghai 200433, China; 16210740012@fudan.edu.cn (Y.P.); 17210740019@fudan.edu.cn (Y.Y.); sunt17@fudan.edu.cn (T.S.); 14210740010@fudan.edu.cn (Y.W.); xieyj@fudan.edu.cn (Y.X.)

**Keywords:** water quality index, trend analysis, influencing factor identification, South-to-North Water Diversion project of China

## Abstract

Xuzhou is the hub city of the east route of China’s South-to-North Water Diversion (SNWD) project and implemented dozens of measures to ensure the water quality security of the water transmission line. In order to detect the effectiveness of water quality improvement measures, the monthly water quality data of five water quality parameters from 2005 to 2015 of six state-controlled monitoring sites in Xuzhou section were selected for analysis. The results showed that the water quality improved from 2.95 in 2005 to 2.74 in 2015, as assessed by the comprehensive water quality identification index (CWQII), and basically reached the Class III standards of China’s Environmental Quality Standard for Surface Water (GB3838-2002) from 2011 to 2015. The trend analysis showed that the decline of ammonia nitrogen (NH_3_-N) was the most obvious among the five water quality parameters. However, the concentrations of phosphorus (TP) showed significant upward trends at three sites. The positive abrupt change of time series of water quality occurred in 2009–2011. The identification of influencing factors of water quality changes by multivariate statistical methods found that the urbanization factor accompanied by a decrease in agricultural nonpoint source pollution emissions and the enhancement of wastewater treatment capacity, the closure of factories with substandard emissions and precipitation were the major influencing factors of most water quality parameters, which confirmed the effectiveness of measures for water quality improvement in Xuzhou.

## 1. Introduction

Interbasin water transfer refers to the construction of water transfer projects across two or more river basins to transfer water resources from the regions with abundant water resources to those in shortage, which is used worldwide to resolve the uneven distribution of water resources among regions [1]. By 2015, more than 160 interbasin water transfer projects had been completed or under construction in about 20 countries and regions, among which the South-to-North Water Diversion (SNWD) project in China is the largest in the world [2]. The SNWD project has a total length of 3833 km trunk canal and is planned to transfer 44.8 billion m^3^ of water per year when fully completed in 2050 [3]. The project has three transfer routes: the east route, the middle route and the west route. The construction of the SNWD project started in late 2002, and the first phase of the east and middle route of the project were completed in 2013 and 2014 [3,4].

As the world’s largest hydraulic project, the SNWD project has caused some controversies for its potential negative environmental impact, such as water pollution and saltwater intrusion in water supply regions [5]. The water transmission line of the east route passes through the Yangtze River Delta and Huaihe Basin, which are seriously polluted. Some researches pointed out that water pollution had become a main constraint to the SNWD project [4].

The east route of the SNWD project passes through three provinces and one municipality (Jiangsu, Shandong, Hebei Provinces, and Tianjin Municipality) and Xuzhou city in Jiangsu Province is a hub city of the east route [6]. Xuzhou is located at the border of Jiangsu Province, which is mainly water supply area, and Shandong province, which is mainly a water use area. It plays an important role in ensuring water quality security for the water use area in next section. However, as a heavy industry base and an important grain production base of China, Xuzhou has a large industrial wastewater discharge and serious problems of agricultural nonpoint source pollution [7,8]. In addition, the acceleration of urbanization in recent years has brought considerable pressure to the water quality in Xuzhou [9].

In order to improve water quality, Xuzhou invested a large amount of funds in pollution control projects and implemented dozens of measures, including sewage diversion projects, sewage treatment plant construction, wetland ecological restoration, and closing down factories with substandard emissions. However, it is still unclear whether these measures have indeed improved the water quality in Xuzhou section of the east route of SNWD. Therefore, it is particularly important to make a comprehensive assessment of water quality over a long period and identify the influencing factors of water quality changes in Xuzhou section.

The water quality index (WQI) is a method summarizing different water quality parameters and transforming massive water quality data into a single number, which can assess water quality in a comprehensive way [10,11]. Since the 1960s, many water quality indexes have been developed to assess water quality for different conditions. Yang et al. [12] used several different methods to assess the water quality of Chaohu Lake basin in China and found that the comprehensive water quality identification index (CWQII) could better assess the water quality compared with the other methods by providing qualitative and quantitative analysis. Because of this advantage, the CWQII method had wide application in the water quality assessment of the lakes and rivers in China, such as Honghu Lake [13], Donghu Lake [14], and Hanjiang River [15].

Trend analysis methods are also used widely in long-term water quality assessment. Since hydrometeorological time series are frequently non-normally distributed, nonparametric methods such as Mann–Kendall test, Sen’s slope estimator, and the sequential Mann–Kendall test are more commonly used in water quality assessment [16]. It is a coherent trend analysis procedure with extensive applications in the field of hydrometeorology, which uses the Mann–Kendall test to determine the trend and trend significance of time series, the Sen’s slope to determine the trend slope, and then the sequential Mann–Kendall test to detect the abrupt change point. For example, Chen et al. [17] applied this trend analysis procedure to the water quality assessment of Fuxian Lake and found that the water quality decreased consistently with an abrupt change point in 2007.

Several research groups studied the relationships between several influencing factors and water quality. Zhao et al. [18] analyzed the impact of landscapes on water quality in a typical headwater catchment of Southeast China by using canonical correlation analysis (CCA) and found that land use patterns in the surrounding areas had an indirect impact on the concentration and migration of pollutants. Scheili et al. [19] studied the impact of climate factors on water quality by using regression analysis. The existing studies mostly focused on the impact of one individual influencing factor on water quality. In fact, the water quality could be affected by several complex factors. However, few studies considered the impact of different types of factors that influenced water quality and identified the major influencing factor.

As for the water quality assessment and trend analysis of the east route of SNWD project, the existing studies mainly focused on the influence of the project on the water quality of the lakes along the water transmission line, such as Nansi Lake [20,21], Hongze Lake [22], and Dongping Lake [23]. However, few studies evaluated the water quality of the water transmission line itself. The only existing study related to the water quality assessment in Xuzhou section of the water transmission line of SNWD project was conducted in 2004; it was found that water quality in three of the six monitoring sites did not meet the Class III standards of China’s Environmental Quality Standard for Surface Water (GB3838-2002) [24]. The investigations of the long-term water quality of Xuzhou section were rarely reported. So, it was difficult to illustrate the water quality trend of the project based on the existing studies. In addition, few studies had combined the water quality trends with local anthropogenic activities and meteorological factors. What are the driving factors of the water quality changes of the project? Are the water quality improvement measures for the SNWD project Xuzhou section effective? These questions still remain unclear.

Considering the above factors and research gaps, a comprehensive assessment and trend analysis of monthly water quality data from 2005 to 2015 of six state-controlled monitoring sites in Xuzhou section of the east route of the SNWD project were conducted in this study by using the comprehensive water quality identification index (CWQII) and nonparametric statistical methods, including Mann–Kendall test, Sen’s slope estimator test, and sequential Mann–Kendall test. In addition, detailed analysis on the identification of influencing factors for water quality changes in Xuzhou section were conducted by using multivariate statistical methods. This work aims to detect the effectiveness of the efforts for water quality improvement and provide targeted suggestions to local government for water quality management in Xuzhou section.

## 2. Materials and Methods

### 2.1. Study Area

Xuzhou city is located in the northwestern part of Jiangsu Province, the southeastern part of the North China Plain and north of the Yangtze River Delta, which is a transition zone between the north and the south of China. The main topography of Xuzhou is plain, accounting for about 90% of the area, with an elevation between 30 and 50 m. Xuzhou has a subhumid warm temperate continental monsoon and four seasons, with an annual average temperature of around 15 °C and an annual average precipitation ranging from 800 to 1200 mm [8]. More than 80% of the precipitation occurs during the wet season (from April to September).

The land use of Xuzhou is divided into six types: agricultural land, woodland, grassland, construction land, water and unused land (the land use maps and proportion of land use types of Xuzhou in 2005 and 2015 can be seen in Figure S1). Among them, agricultural land accounts for the largest area of Xuzhou’s land area (more than 60%). The proportion of agricultural land in Xuzhou decreased by 2.91% and the proportion of construction land in Xuzhou increased by 3.03% from 2005 to 2015.

### 2.2. Data Collection

**Figure 1:**
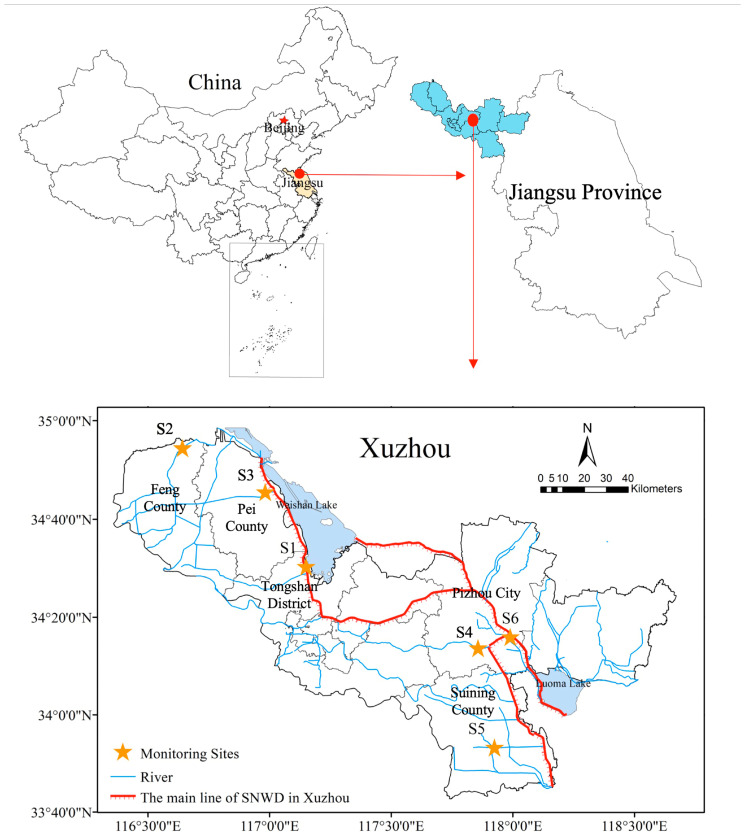
Map of the state-controlled monitoring sites in Xuzhou section.

To ensure the water quality security, six state-controlled monitoring sites, including Linjiaba (S1), Shazhuangqiao (S2), Lijiqiao (S3), Danjizha (S4), Shajixizha (S5), and Zhanglou (S6), were set in Xuzhou section (Figure 1). Among the six monitoring sites, S1 and S6 are located in the trunk stream of Beijing–Hangzhou grand canal, which is the main water transmission line of the SNWD project. The other four sites are located in the tributary of Beijing–Hangzhou grand canal. As for the location at the county level, S1 is located in Tongshan District, S2 is located in Feng County, S3 is located in Pei County, S4 and S6 are located in Pizhou City (county-level), and S5 is located in Suining County.

In this study, five water quality parameters, including dissolved oxygen (DO), chemical oxygen demand by KMnO_4_ (COD_Mn_), five-day biochemical oxygen demand (BOD_5_), ammonia nitrogen (NH_3_-N) and total phosphorus (TP), were selected for water quality analysis. The first three parameters are related to organic pollutants, and the latter two parameters are related to nutrients. Monthly monitoring data of water quality from 2005 to 2015 of the six state-controlled monitoring sites were selected for analysis, including nine years of the construction period (2005–2013) and two years of the operation period (2014–2015). There were three parallel samples for each sampling. The water quality data were collected from Xuzhou Environmental Monitoring Station. The land use data were derived from National Earth System Science Data Sharing Infrastructure of China (http://www.geodata.cn). The meteorological, socioeconomic, and pollution data were collected from the statistical yearbook of Xuzhou, China, from 2005 to 2015 [25].

### 2.3. Analysis Methods

#### 2.3.1. Comprehensive Water Quality Identification Index (CWQII)

The comprehensive water quality identification index (CWQII) developed by Xu [26] is widely used in the water quality assessment in China [12,14]. This method has several advantages: it can perform both qualitative and quantitative evaluation of water quality; it can be used not only to evaluate the water quality of different sections in the same river, but also to compare the water quality in different rivers; it can avoid the influence of an individual water quality indicator, so that the integrated condition of the water quality can be reflected reasonably; and it can not only divide the water quality into different categories, but also compare the water quality of the same category [12]. The calculation of CWQII is given as follows.

CWQII consists of an integer and two or three digits:

(1) $$CWQII=X_{1}\cdot X_{2}X_{3}X_{4}$$

where $X_{1}$ represents the composite water quality grade and $X_{2}$ represents the position of water quality in the range of Class $X_{1}$. The following equation is used to calculate $X_{1}\cdot X_{2}$:

(2) $$X_{1}\cdot X_{2}=\frac{1}{n}\overset{n}{\underset{i=1}{\sum }}P_{i}=\frac{1}{n}\overset{n}{\underset{i=1}{\sum }}x_{1}\cdot x_{2\;}$$

where *n* is the number of water quality parameters in water quality assessment; $P_{i}$ is the single factor water quality identification index (SFWQII) of the $i^{th}$ water quality parameter ($i^{th}$ refers to the order of the parameters for the calculation of SFWQII: 1st, 2nd, 3rd, 4th…); $x_{1}$ represents the water quality grade of the single water quality parameter; and $x_{2}$ represents the water quality position of the single parameter in the range of Class $x_{1}$.

If the water quality parameter concentration is between Class I to Class V according to China’s Environmental Quality Standard for Surface Water (GB 3838-2002) (the standard values for Class I to Class V of the five main water quality parameters are shown in Table S1), $x_{1}$ is achieved by comparing the value with the standard. For example, if the water quality parameter concentration is below the standard of Class I, then $x_{1}\;$= 1; if the concentration is between the standard of Class I and Class II, then $x_{1}\;$= 2, and so on. The index $x_{2}$ is calculated by the following equation (except DO):

(3) $$x_{2}=\frac{c_{i}-c_{ikMin}}{c_{ikMax}-c_{ikMin}}\times 10$$

where $c_{i}$ is the measured concentration of the $i_{th}$ water quality parameter, $c_{ikMin}$ is the lower limit value of the Class *k* range for the $i^{th}$ water quality parameter, and $c_{ikMax}$ is the upper limit value of the Class *k* range for the $i^{th}\;$ water quality parameter. The result keeps one significant figure.

As for the index of dissolved oxygen (DO), the equation is as follows:

(4) $$x_{2}=\frac{c_{ikMax}-c_{i}}{c_{ikMax}-c_{ikMin}}\times 10$$

The result also keeps one significant figure.

If the water quality parameter concentration is beyond the standard of Class V, then $x_{1}\cdot x_{2}$ is calculated by the following equation (except DO):

(5) $$x_{1}\cdot x_{2}=6+\frac{c_{i}-c_{i5Max}}{c_{i5Max}}$$

The result keeps one significant figure after the decimal point.

As for the index of DO,

(6) $$x_{1}\cdot x_{2}=6+\frac{c_{i5Min}-c_{i}}{c_{i5Min}}\times m$$

where *m* is correction factor, generally *m* = 4. The result keeps one significant figure after the decimal point.

$X_{3}$ is the number of the water quality parameters that are worse than the standards of environmental functions of surface water (EFSW). $X_{4}$ is the result of the composite water quality compared with EFSW. If $X_{4\;}$= 0, then the composite water quality achieves EFSW; otherwise, $X_{4}=X_{1}-f\left(X_{4}\neq 0\right)$, or $X_{4}=X_{1}-f-1\left(X_{4}=0\right)$, where *f* represents the grade of EFSW for the study area.

In this study, some of the 24 water quality parameters routinely monitored were not detected in several sites, so $X_{3}$ is difficult to evaluate across sites. Therefore, the value of $X_{1}\cdot X_{2}$ is regarded as CWQII in this study. Undetected values were not included in the calculation of CWQII.

#### 2.3.2. Trend Analysis Methods

Trend analysis from 2005 to 2015 was conducted for the five water quality parameters selected. Three nonparametric statistical methods, Mann–Kendall test, Sen’s slope estimator test, and sequential Mann–Kendall method, were selected for trend analysis in this study. The process of trend analysis was completed by MATLAB_R2016a (The MathWorks, Natick, MS, USA).

The Mann–Kendall test developed by Mann and Kendall [27] is a nonparametric rank-based test method and has been used widely to assess the significance of trend in hydrometeorological time series, such as water quality, temperature, and precipitation [28,29]. The main reason for using nonparametric statistical tests is that compared with parametric statistical tests like linear regression, is that the nonparametric tests are more suitable for non-normally distributed data and censored data, which occur frequently in hydrometeorological time series [16]. In this study, the Mann–Kendall test was used to judge whether the trend of the time series of water quality parameters was increasing or decreasing, and to determine if the trend was statistically significant.

Sen developed the nonparametric procedure for estimating the slope of trend [30]. Sen’s slope estimator is always combined with the Mann–Kendall test for trend analysis of the hydrometeorological time series [28,29]. Since there are different standards for each water quality parameter, the results of Sen’s slope can only compare the trend level of the same parameter. In order to compare the trend level of different parameters, the index *L_n_* (level of tendency) was introduced in this study. *L_n_* was calculated as:

*L_n_* = *Q_n_*/*C_n_* (7)

where Q_n_ is the result of Sen’s slope, C_n_ is the Class III standard value of China’s Environmental Quality Standard for Surface Water (GB3838-2002) for each parameter (DO, 5 mg/L; COD_Mn_, 6 mg/L; BOD_5_, 4 mg/L; NH_3_-N, 1 mg/L; TP: 0.2 mg/L). The larger the absolute value of L_n_, the more obvious the upward or downward trend.

The sequential Mann–Kendall test can be used to identify the abrupt change point of the time series with statistically significant trends. It is a sequential procedure of forward and backward analyses of the Mann–Kendall test. If the two series (forward and backward) cross and then diverge from each other for a longer period of time, the year of crossing represents the year of abrupt change [17]. This method has also been widely used in the analysis of hydrometeorological time series [31,32]. The detailed calculation steps can be found in Sayemuzzaman and Jha [33].

It is worth noting that the time series have to be serially independent before the application of the trend analysis methods. The process of prewhitening was applied to remove the serial correlation effect before the trend analysis methods. The specific steps can be found in Gocic and Trajkovic (2013) [34]. The process of prewhitening was conducted in EViews 6.0 (Quantitative Micro Software, Los Angeles, CA, USA).

#### 2.3.3. Multivariate Statistical Methods

Multivariate statistical methods have been widely used in water quality assessment [35,36]. In this study, the Pearson correlation analysis was applied to identify the relationship between water quality parameters and their influencing factors. Principal component analysis (PCA) was used to reduce the dimension of the raw data by eliminating overlapping information and then classifying the influencing factors into independent groups. The principal component regression was carried out to determine the relationship of water quality parameters and the principal components of influencing factors. The multivariate statistical analysis was conducted by IBM SPSS Statistics 21.0. (IBM, Armonk, NY, USA).

## 3. Results and Discussion

### 3.1. Characteristics of Water Quality Parameters in Xuzhou Section

**Figure 2:**
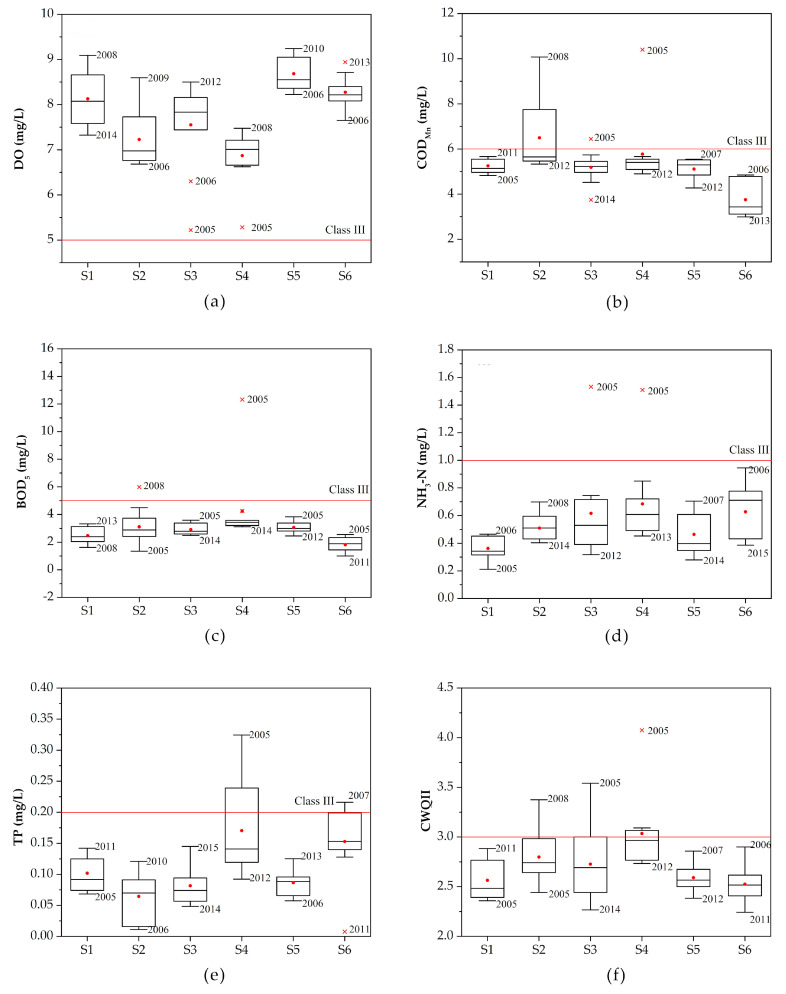
Box plots of (**a**) DO, (**b**) COD_Mn_, (**c**) BOD_5_, (**d**) NH_3_-N, (**e**) TP, and (**f**) CWQII (comprehensive water quality identification index) for the six sites in Xuzhou section. The year when the maximum and minimum values of each water quality parameter occurred are marked in the box plot.

The characteristics of water quality parameters in Xuzhou section are shown in Figure 2. S4 had the highest concentrations of almost all the five parameters (DO was the lowest) among the six sites, indicating that the water quality in S4 was the poorest. In addition, the concentrations of organic pollutants in S2 and nutrients in S6 were relatively higher than those of the other sites.

The maximum values of DO and the minimum values of the other four parameters were defined as the best values, and the minimum values of DO and the maximum values of the other four were defined as the worst values. As shown in Figure 2, most of the worst values occurred before 2010. S4 was the most typical site with all the five worst values occurred in 2005. As for the best values of each parameter, most of them occurred after 2010, which showed that most sites have a positive trend of water quality. S1 was the only special one with the best values occurring before 2010 and the worst values occurring after 2010, indicating that the water quality in S1 may have a negative trend.

According to the *Pollution Control Planning for the East Route of the South-to-North Water Transfer Project* [37], the water quality parameters in Xuzhou section had to meet the demand of China’s Environmental Quality Standard for Surface Water (GB3838-2002) Class III (DO: >5 mg/L, COD_Mn_: <6 mg/L, BOD_5_: <4 mg/L, NH_3_-N: <1 mg/L, TP: <0.2 mg/L). The qualified rate for Class III of each water quality parameter refers to the proportion of the samples that meet the Class III standard for each parameter. It was calculated as:

(8) $$QR_{i}\;=\frac{n_{i}}{n_{total}}\times 100\%$$

where $QR_{i}$ is the qualified rate for Class III of each water quality parameter, $n_{i}$ is the number of the samples that meet the Class III standard for each parameter, and $n_{total}$ is the number of the total samples.

**Figure 3:**
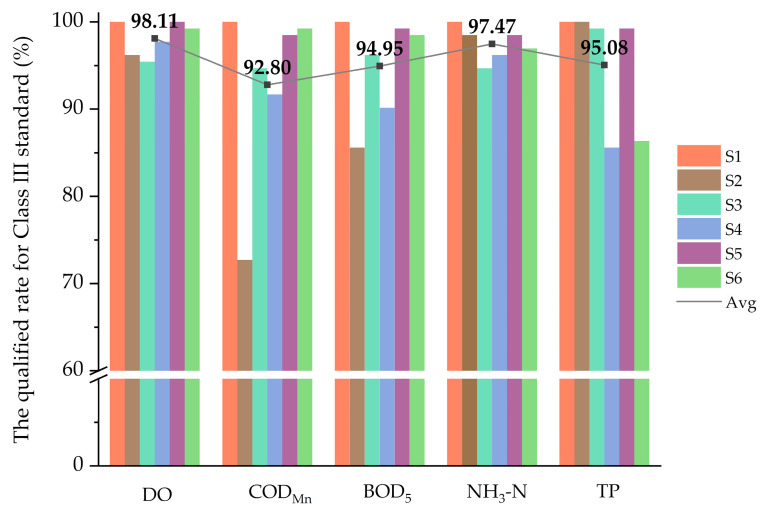
The qualified rate for Class III standard of each parameter in six sites.

The qualified rate for Class III of each parameter followed the order of COD_Mn_ (92.80%) < BOD_5_ (94.95%) < TP (95.08%) < NH_3_-N (97.47%) < DO (98.11%) (Figure 3). The compliance rate of COD_Mn_ was relatively low, mainly because that the low compliance rate of COD_Mn_ in S2 (72.73%) pulled down the average value. The water quality in S1 maintained a good level with all the 5 parameters reaching Class III from beginning to end.

### 3.2. Water Quality Assessment by Using CWQII

The CWQII method was used to make a more overall assessment of the pollution characteristics of the water environment in the study area. As for spatial distribution, the average annual CWQII of the six sites followed the order of S6 (2.53) < S1 (2.56) < S5 (2.59) < S3 (2.73) < S2 (2.80) < S4 (3.03) (Figure 2). In general, water quality in the sites located in the main line of water transmission was better than the sites located in tributaries. It may be due to the fact that most of the rivers in Xuzhou lack natural water supply other than rainfall. As a result, the river flowed slowly and had poor self-cleaning ability. It was difficult to dilute and diffuse sewage into the river, thus easily causing pollution [38]. S4, which was relatively far from the main line of water transmission was the site with the worst water quality and also the only site that exceeded 3.0 assessed by CWQII. However, the coefficient variation of S4 was also the largest among all the six sites, indicating that the water quality in S4 fluctuated greatly.

**Figure 4:**
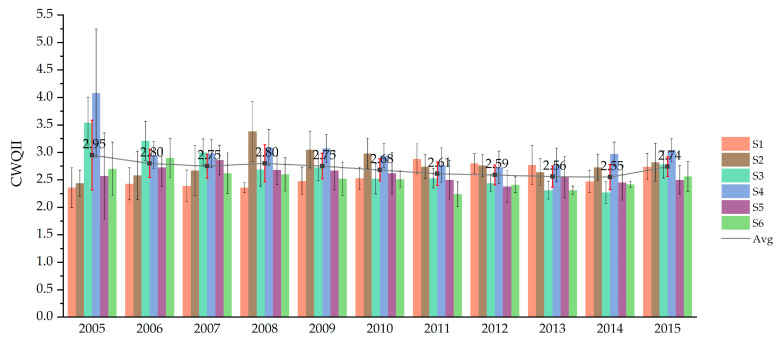
The yearly CWQII of each site in Xuzhou section. The standard deviation error bars were added in the figure. The two decimal places of the CWQII only represented the calculation results of mean values, and the second decimal place was irrelevant to the significance of X3 in CWQII.

As for temporal variation, the annual average CWQII showed an overall downward trend from 2.95 in 2005 to 2.55 in 2014, with a decrease of 13.6% (Figure 4). The first phase of the east route of the SNWD project started water supply in 2013, so there was no obvious deterioration of water quality during the transition from construction period to operation period. However, the water quality had a decline in 2015, with CWQII increasing to 2.74. The CWQII in S3 and S4 in 2005 were obviously higher than that in other sites, reaching 3.54 and 4.08. But the water quality improved a lot in S3 and S4, with CWQII basically dropping below 3.0 since 2010. The CWQII of S2 fluctuated obviously, showing an upward trend from 2005 to 2008, and then beginning to decline. The CWQII in S1 was at a very low level in 2005 but had an upward trend since then. The results of CWQII in Xuzhou section are compared with other studies in Table S2. The CWQII in Xuzhou section was relatively lower than that in other areas but had more fluctuations in the first half of the study period (2005–2010). In the second half (2011–2015), the CWQII in Xuzhou section was further reduced and also became more stable.

**Figure 5:**
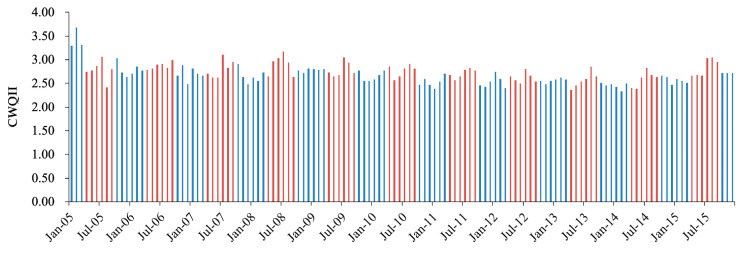
Temporal variation of CWQII in Xuzhou section from 2005 to 2015. The wet seasons (from April to September) were marked in red, and the dry seasons (from October to March in next year) were marked in blue.

Figure 5 shows the time profile of the fluctuation of monthly CWQII from which the seasonal changes of water quality can be seen. The CWQII in the first three months of 2005 was quite high and exceeded 3.0. Specifically for each parameter, the concentrations of COD_Mn_ and BOD_5_ reached 44.3 mg/L and 78.6 mg/L in February 2005, which was 7 times and 20 times higher than Class III of water standards. It might be due to serious pollution incidents. As for seasonal changes, the CWQII during wet seasons (from April to September, 2.75) from 2006 to 2009 were slightly higher than that in dry seasons (from October to March in the next year, 2.66). In particular, the CWQII was obviously higher in July and August (2.92 and 2.81, respectively) than in other months. An independent *t*-test was applied for CWQII in wet and dry seasons and showed a significant difference (*p* < 0.05) between two seasons. In addition, the suspected pollution incidents in the first three months of 2005 (dry season) might have reduced the gap between wet and dry seasons. The difference would be even greater if the data of the first three months of 2005 were excluded (2.62 in dry seasons and 2.75 in wet seasons).

The seasonal variations in Xuzhou section may be due to the differences in precipitation between wet and dry seasons. Precipitation has a dual effect on water quality. On the one hand, the dilution effect by high river flows in wet seasons may lead to relatively lower concentrations of pollutants in wet seasons [39]. On the other hand, high intensity and long-term rainfall would wash a large number of organic and nutrient pollutants into water bodies through surface runoff and underground runoff, resulting in serious nonpoint source pollution, especially in agricultural land [40]. In addition, flooding caused by heavy precipitation could carry a large amount of surface sediments into the water, which would cause the resuspension of sediments in water and further affect the transport and transformation of pollutants [41]. The water quality in wet seasons in Xuzhou section was significantly higher than that in dry seasons, indicating that the pollutant-carrying effect of rainfall was greater than the dilution effect and that the agricultural nonpoint source pollution might be serious in Xuzhou section.

### 3.3. Trend Analysis of Water Quality in Xuzhou Section

Trend analysis methods, including the Mann–Kendall test and Sen’s slope, were used in this study to show in a scientific way the trends of water quality parameters for each site during the period 2005–2015. Table 1 shows the results of these two methods. The significant upward trend of DO and the downward trend of other indicators were defined as positive trends. On the contrary, the significant downward trend of DO and the upward trend of other indicators were defined as negative trends. Except for TP, none of the other five indicators showed significant negative trends at the six sites. NH_3_-N at the four sites and COD_Mn_ at three sites showed a significant positive trend (95% confidence). Among them, COD_Mn_ of S3 and S5 and NH_3_-N of S3 and S6 showed a very significant positive trend (99% confidence). The positive trends of COD_Mn_ and NH_3_-N were more obvious than other parameters. However, the trends of TP had obvious differences in each site. The TP of two sites (S3 and S4) showed a significant positive trend, while three sites (S1, S2, and S5) showed a significant negative trend.

In terms of spatial differences, S3 had the most obvious water quality improvement with all the five water quality parameters and CWQII showing significant positive trends. The water quality in S4, which was the poorest initially, did not show a desired improvement, with only two parameters showing significant positive trends. As for S1 and S2, there was no parameter showing significance positive trend, and TP in the two sites showed significance negative trends. It could be concluded that S3 achieved the best improvement of water quality among the six sites, while S1 and S2 had little progress in water quality.

**Figure 6:**
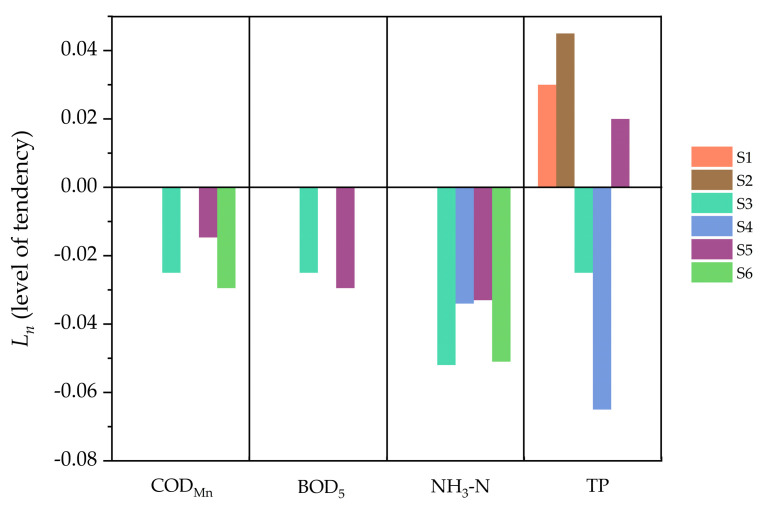
The trend level of each water quality parameter in each site.

The index *L_n_* (level of tendency) was calculated to compare the trend level of each parameter. Because the increase of DO represents the improvement of water quality, which is opposite from the other four parameters, DO was not included in the calculation of *L_n_*. Figure 6 shows the values of *L_n_* for the four parameters at each site (only time series with significant trends are shown). Among all four parameters, the values of *L_n_* of NH_3_-N were the lowest below zero, which indicated that the decline of NH_3_-N was the most obvious and the emission reduction of NH_3_-N had achieved great success.

**Figure 7:**
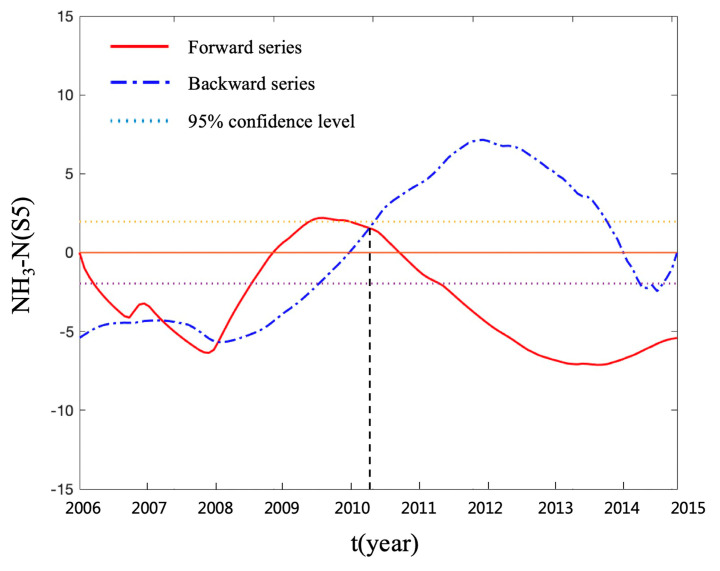
The result of the sequential Mann–Kendall test for the time series of NH_3_-N in S5.

The sequential Mann–Kendall test was used to identify the abrupt change point of the time series of each parameter. The process of prewhitening was applied to remove the serial correlation effect before the sequential Mann–Kendall test [34]. The twelve-month rolling average data were used to avoid the impacts of extreme values in the time series [31]. The complete results of the sequential Mann–Kendall test can be seen in Figure S2. As an example, Figure 7 shows the result of the sequential Mann–Kendall test for the time series of NH_3_-N in S5. The intersection of the two curves corresponds to the abscissa of 2010 and the ordinate is within ±1.96 (95% confidence level), indicating that there was an abrupt change in 2010. Table 2 shows the abrupt change years of the time series for the five parameters and CWQII. It was found that the time series of NH_3_-N in S3, S4, and S5, and TP in S2 had significant abrupt change points. The positive abrupt change points of time series occurred in 2009–2011 and they all belonged to the time series of NH_3_-N.

### 3.4. Identification of Influencing Factors of Water Quality Changes in Xuzhou Section

**Figure 8:**
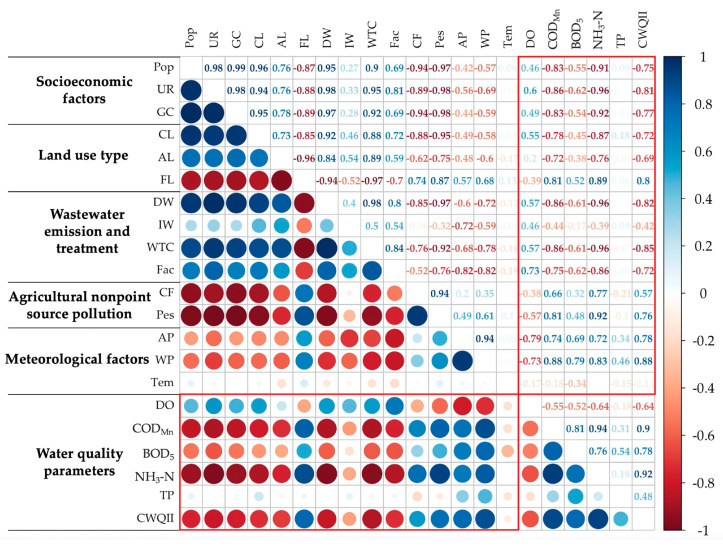
Pearson correlation coefficients of water quality parameters and their influencing factors in Xuzhou section. The full names of indicators are as follows: Pop–population; UR–urbanization rate; GC–GDP per capita; CL–construction land area; AL–agricultural land area; FL–forest land area; DW–domestic wastewater discharge; IW–industrial wastewater discharge; WTC–wastewater treatment capacity; Fac–number of factories; CF–chemical fertilizer application; Pes–pesticide application; AP–annual precipitation; WP–wet season precipitation; Tem–temperature. If the absolute value of correlation coefficient exceeds 0.6/0.75, it means that the correlation is statistically significant at the 95%/99% confidence level.

There are several factors causing the changes of water quality, including socioeconomic development, land use patterns, industrial activities, agricultural activities, wastewater treatment, and meteorological factors [9,42]. In this study, 15 factors of different categories were selected to analyze their relationships with water quality, including population, urbanization rate, GDP per capita, construction land area, agricultural land area, forest land area, domestic wastewater discharge, industrial wastewater discharge, wastewater treatment capacity, number of factories, chemical fertilizer application, pesticide application, annual precipitation, wet season precipitation, and temperature. The data of the influencing factors were collected from the Xuzhou statistical yearbook. After confirming that all of the indicators were normally distributed, Pearson correlation analysis was conducted to study the relationships among the 15 influencing factors and 6 water quality parameters (Figure 8).

**Figure 9:**
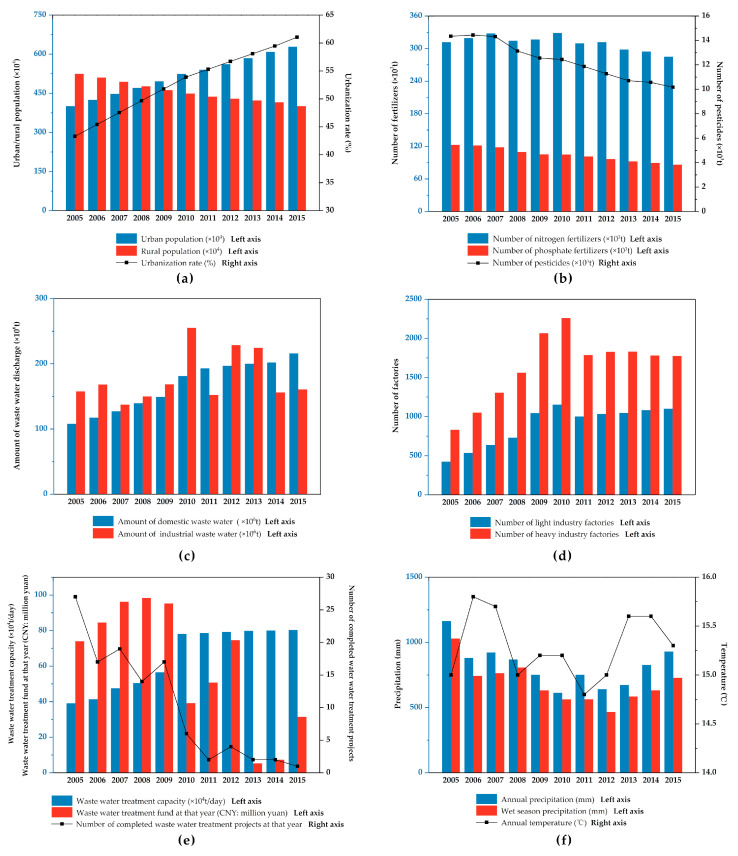
The situation of (**a**) socioeconomic factors, (**b**) chemical fertilizer and pesticide emission, (**c**) waste water discharge, (**d**) number of factories, (**e**) waste water treatment, and (**f**) meteorological factors for Xuzhou from 2005 to 2015.

There were significant correlations between water quality parameters and most anthropogenic factors. Three socioeconomic factors, population, urbanization rate, and GDP per capita, had a significant negative correlation with water pollutant concentrations. While urbanization and population growth led to an increase in domestic waste water discharge (Figure 9c), at the same time agricultural modernization resulted in an obvious decline in application of chemical fertilizers and pesticides (Figure 9b). The application of nitrogen fertilizers, phosphate fertilizers, and pesticides decreased by 8.6%, 29.8%, and 29.1%, respectively, over the 11 years. Meanwhile, wastewater treatment capacity had doubled during this period. The reduction of agricultural nonpoint pollution and the increase in wastewater treatment capacity could lead to the improvement of water quality [43].

Xuzhou invested heavily in wastewater treatment from 2005 to 2009, with the completion of 94 wastewater treatment projects in five years. The water quality capacity of Xuzhou had a significant increase in 2010 and then changed little from 2011 to 2015 (Figure 9e). After 2010, the funds and completed projects for sewage treatment also had an obvious decline. However, water quality remained stable since then, indicating that the wastewater treatment capacity had basically met the needs of Xuzhou.

There were also significant correlations between industrial activities and water quality. One of the most important measures to control water pollution in Xuzhou was the closure of a large number of factories that did not discharge pollutants up to standard, especially in heavy industry (Figure 9d). In 2011, 13.1% of light industry factories and 20.9% of heavy industry factories closed, resulting in a decrease in industrial wastewater discharge (Figure 9b). The shutdown of factories did have a positive impact on the improvement of water quality. The abrupt change point analysis of water quality pointed out that the concentrations of NH_3_-N had an abrupt decline between 2009 and 2011 in several sites, which may due to the evident increase of wastewater treatment capacity in 2010 and decrease of number of factories in 2011.

Among the meteorological factors, there was a significant correlation between precipitation and water quality, especially wet season precipitation, which was consistent with many studies [44,45,46]. The results of seasonal analysis also found that the water quality was significantly worse in wet seasons than in dry seasons. Nonpoint agricultural source pollution is greatly affected by precipitation and runoff processes [40,47]. Therefore, although the application of pesticides and fertilizers in Xuzhou had declined since 2005, nonpoint agricultural source pollution was still a significant restriction for water quality in Xuzhou. There was a clear decline in water quality in 2015 (Figure 4), which may have been due to the fact that the precipitation in 2015 was obviously higher than previous years (12.29% more annual precipitation and 14.79% more wet season precipitation compared to 2014) (Figure 9f). The precipitation in 2005–2008 in Xuzhou was much higher than the following years, which could contribute to the relatively poor water quality during that period.

No significant correlation was found between temperature and water quality. Some studies had pointed out that higher temperature may lead to temperature stratification in the water, which could promote the release of pollutants from bottom sediment into the water [48,49]. In addition, temperature is also an important factor in eutrophication [50]. However, the annual temperature of Xuzhou remained stable during the monitoring period, with an annual average temperature range of no more than 1 degree centigrade. Therefore, temperature was not the dominant influencing factor of water quality.

It is worth noting that the concentrations of TP had no significant correlation with any of the selected influencing factors and other water quality parameters. So, it was assumed that the concentrations of TP were primarily derived from the release of endogenous phosphorus from the sediments rather than from pollutants discharged into the water. Sediments are important reservoirs of nutrients in rivers. Nutrients accumulated in sediments would gradually release under appropriate conditions, leading to increased nutrient pollution in water [51]. The release of phosphorus from sediments is influenced by a complex set of factors, including dissolved oxygen concentration, pH, water temperature, water disturbance, biological factors such as algal and microorganisms, phosphorus concentration in the overlying water, sediment composition, etc. [52,53,54]. Sampling and measurement of the sediments is required to analyze the specific mechanism of the release of phosphorus in Xuzhou section.

Principal component analysis (PCA) was applied to reduce the dimension of the raw data by eliminating overlapping information and then classify the influencing factors of water quality. Due to the strong correlation between indicators of the same category, some indicators were not included for PCA. The urbanization rate was selected as a proxy for socioeconomic factors, the area of forest land as a proxy for land use factors, and wet season precipitation as a proxy for meteorological factors. The KMO (Kaiser–Meyer–Olkin) test and Bartlett’s test were conducted for data reliability. The PCA results are shown in Table 3. Two components with eigenvalues > 1 explained 89.565% of the total variance. The first principal component (PC1), accounting for the most variance (72.782%), had high loadings on the indicators of urbanization rate, application of chemical fertilizers and pesticides, domestic wastewater discharge, area of forest land, and wastewater treatment capacity. PC1 represented the urbanization factor. The increased urbanization in Xuzhou was accompanied by an increase in domestic wastewater discharge, a decrease in agricultural nonpoint source pollution, and the enhancement of wastewater treatment capacity. The second principal component (PC2), accounting for 16.783% of variance, had high loadings on the indicators of wet season precipitation, number of factories, and industrial wastewater discharge. It represented the impact of the meteorological factor and industrial activities.

Principal component regression was conducted with the factor scores of two principal components as independent variables and water quality parameters as dependent variables. The results are shown in Table 4. The regressions for COD_Mn_, NH_3_-N, and CWQII were significant at the 99% confidence level, and the regressions for DO and BOD_5_ were significant at the 90% confidence level. It indicated that the urbanization factor as well as precipitation and industrial activities were important factors influencing the change of water quality indicators other than TP. COD_Mn_ and NH_3_-N were more significantly affected by these factors.

Each site in Xuzhou section was discussed separately for the analysis of spatial difference in water quality changes. Since the six state-controlled monitoring sites in Xuzhou section are almost evenly distributed in the five districts and counties of Xuzhou, the situation of the indicators listed in Table 5 of the districts and counties where the sites are located could roughly represent the situation of each site. According to the trend analysis of water quality, S3 was the site with the most obvious water quality improvement and the concentrations of NH_3_-N in S3 had an abrupt change in 2011. It can be seen from Table 5 that the reduction of chemical fertilizer application and the increase of the domestic waste water treatment rate of Pei County, where S3 was located, were the highest among all the districts and counties. In addition, Pei County shut down 34.6% of factories in 2011 (also the most in Xuzhou), which could contribute to the abrupt decline of NH_3_-N in 2011. While, Tongshan District and Feng County, where S1 and S2 are located, did not have evident progress on these indicators compared with other areas, which may result in the nonideal improvement of water quality in S1 and S2. In the first half of the research period (2005–2010), the average domestic sewage treatment rate of Pizhou City, where S4 and S6 are located, was only 67.5%, which was the lowest among all the districts and counties, resulting in the poor initial water quality of the two sites. However, the number of factories in Pizhou City decreased by 16.4% in 2011, second only to Pei County, which might contribute to the water quality improvement in S4 and S6.

Based on the results of identification of water quality changes in Xuzhou section, targeted measures need to be implemented. Firstly, further control should be strengthened on agricultural nonpoint source pollution. In addition to reducing emissions of pollutants at the source, it is also necessary to intercept pollutants in the transmission pathway and carry out ecological restoration of agricultural ecosystem [55,56]. Some ecological engineering techniques like ecological ditches, constructed wetlands, and vegetated filter strips should be applied in Xuzhou section [57,58]. Secondly, measures are needed to control the release of phosphorus from sediments to water. Ecological dredging and establishment of constructed wetlands are effective means to reduce the release of endogenous phosphorus [59].

To sum up, in the process of identifying influencing factors of water changes in this study, the first step was to select 15 factors of different categories to analyze their relationships with water quality. Next, the factors with high correlations with water quality were classified into two categories by principal component analysis: PC1 (urbanization factor) and PC2 (meteorological factor and industrial activities). Finally, the principal component regression was conducted with the factor scores of two principal components as independent variables and water quality parameters as dependent variables. The two principal components were found to have strong correlations with most water quality parameters, which confirmed the effectiveness of water quality improvement measurements in Xuzhou section. Most existing studies focused on the impact of individual influencing factor on water quality. In fact, the water quality could be affected by several complex factors and it is important to select the major influencing factors. This study tried to use several multivariate statistical methods to identify the influencing factors of water quality changes in Xuzhou section and obtained reasonable results. It might provide useful experience for the identification of influencing factors of water changes in other areas.

## 4. Conclusions

This study conducted a comprehensive assessment and trend analysis of water quality for six monitoring sites in the Xuzhou section of the east route of the SNWD Project from 2005 to 2015 and identified the influencing factors of water quality changes. The main conclusions follow:

1. The water quality in the study area had greatly improved during the monitoring period assessed by CWQII and basically reached Class III of China’s Environmental Quality Standard for Surface Water (GB3838-2002) from 2011 on. In terms of seasonal variation, the water quality in wet season (2.75) was worse than that in dry season (2.66), mainly due to the more serious agricultural nonpoint source pollution in wet season.
2. The trend analysis showed that the concentrations of NH_3_-N had a most obvious decline among the five parameters with four sites showing significant positive trends. However, the concentrations of TP showed significant upward trends in three sites. For each monitoring site, Lijiqiao (S3) had the most obvious improvement in water quality, with all five parameters showing significant positive trends during the 11 years. In contrast, Linjiaba (S1) and Shazhuangqiao (S2) had little progress in water quality. The positive abrupt change points of time series occurred in 2009-2011, and they all belonged to the time series of NH_3_-N.
3. The identification of influencing factors of water quality changes found that the urbanization factor, the closure of factories with substandard emissions, and precipitation were the major influencing factors of most water quality parameters, especially COD_Mn_ and NH_3_-N. The concentrations of TP were irrelevant with anthropogenic activities and meteorological factors, and were assumed to be primarily derived from the release of endogenous phosphorus from the sediments rather than from pollutants discharged into the water. Overall, the water quality improvement measures in Xuzhou section had been effective.

## Figures and Tables

**Table 1:** Results of trend analysis for five water quality parameters and CWQII over the period 2005–2015.

Site	Index	DO	COD_Mn_	BOD_5_	NH_3_-N	TP	CWQII
S1	*Z*	−0.311	0.779	1.246	0.311	2.180(−) *	1.868
	*Q*	−0.033	0.042	0.074	0.002	0.006(−) *	0.043
S2	*Z*	0	−1.868	0.779	−0.311	2.024(−) *	0.623
	*Q*	−0.002	−0.131	0.060	−0.006	0.009(−) *	0.018
S3	*Z*	2.491(+) *	−3.114(+) **	−2.491(+) *	−2.803(+) **	−2.180(+) *	−2.803(+) **
	*Q*	0.162(+) *	−0.150(+) **	−0.100(+) *	−0.052(+) **	−0.005(+) *	−0.104 **
S4	*Z*	−0.156	−0.623	−1.557	−2.336(+) *	−2.024(+) *	−1.401
	*Q*	−0.008	−0.043	−0.056	−0.034(+) *	−0.013(+) *	−0.026
S5	*Z*	0.779	−2.647(+) **	−3.425(+) **	−2.180(+) *	2.647(−) **	−2.413(+) *
	*Q*	0.025	−0.088(+) **	−0.118(+) **	−0.033(+) *	0.004(−) **	−0.028(+) *
S6	*Z*	1.713	−2.336(+) *	0.156	−3.114(+) **	−0.311	−2.336(+) *
	*Q*	0.033	−0.177(+) *	0.046	−0.051(+) **	−0.001	−0.042(+) *

*Z*—results of Mann–Kendall test, *Q*—results of Sen’s slope; “+” indicates a significant positive trend, “−” indicates a significant negative trend; ** *p* < 0.01, * *p* < 0.05.

**Table 2:** The year of abrupt change of the five water quality parameters in each site.

Site	DO	COD_Mn_	BOD_5_	NH_3_-N	TP	CWQII
S1	--	--	--	--	2008 (+)	--
S2	--	--	--	--	2008 (+) *	--
S3	2008 (+)	2011 (−)	2008 (−)	2011 (−) *	2008 (−)	2008 (−)
S4	--	--	--	2009 (−) *	2010 (−)	--
S5	--	2010 (−)	2008 (−)	2010 (−) *	2010 (+)	2010 (−)
S6	--	2009 (−)	--	2012 (−)	--	2008 (−)

Only the results of the sequential Mann–Kendall test of time series with significant trends are shown in the table; “+” indicates an ascending trend and “−” indicates a descending trend; * *p <* 0.05.

**Table 3:** The component matrix of the influencing factors of water quality in Xuzhou section.

Variables	PC1	PC2
Pesticide application (Pes)	−0.902	
Chemical fertilizer application (CF)	−0.898	
Urbanization rate (UR)	0.868	
Domestic wastewater discharge (DW)	0.838	
Forest land (FL)	−0.758	
Wastewater treatment capacity (WTC)	0.746	
Wet season precipitation (WP)		−0.879
Number of factories (Fac)		0.810
Industrial wastewater discharge (IW)		0.800
Eigenvalues	6.550	1.510
% of Variance	72.782	16.783
Cumulative %	72.782	89.565

KMO (Kaiser–Meyer–Olkin) test value is 0.613 and Bartlett’s test value is 158.125 at the significance level of *p* < 0.01.

**Table 4:** Regression estimations of the water quality parameters.

Parameter	Standardized Regression Coefficient		R-Squared
	PC1	PC2	
**DO**	0.250	0.664 *	0.504 ^+^
**COD_Mn_**	−0.638 **	−0.628 **	0.802 **
**BOD_5_**	−0.336 ^+^	−0.556 *	0.422 ^+^
**NH_3_-N**	−0.740 **	−0.625 **	0.938 **
**TP**	0.164	−0.234	0.082
**CWQII**	−0.553 *	−0.661 **	0.744 **

** *p* < 0.01, * *p* < 0.05, ^+^ *p* < 0.1.

**Table 5:** The influencing factors of each district and county in Xuzhou.

Region	Section	Period	CFP	PP	DWR	IWR	Fac
**Tongshan District**		First half	0.990	0.013	80.6%	98.9%	535
	S1	Second half	0.939	0.011	82.6%	99.2%	463
		Change rate	−5.2%	−15.4%	+2.0%	+0.3%	−13.5%
**Feng County**		First half	1.549	0.033	78.4%	95.2%	244
	S2	Second half	1.489	0.027	83.2%	97.6%	235
		Change rate	−3.9%	−18.2%	+4.8%	+2.4%	−3.7%
**Pei County**		First half	1.423	0.025	83.4%	97.8%	710
	S3	Second half	1.310	0.021	88.6%	98.9%	464
		Change rate	−7.9%	−16.0%	+5.2%	+1.1%	−34.6%
**Pizhou City**		First half	1.384	0.025	67.5%	96.2%	645
	S4, S6	Second half	1.343	0.021	72.1%	97.6%	539
		Change rate	−3.0%	−16.0%	+4.6%	+1.4%	−16.4%
**Suining County**		First half	1.079	0.015	73.2%	99.0%	314
	S5	Second half	1.024	0.012	78.6%	99.4%	271
		Change rate	−5.1%	−20.0%	+5.4%	+0.4%	−13.7%

The full names of indicators in the first line are as follows: CFP–amount of chemical fertilizers applied per hectare (unit: t/ha), PP–amount of pesticides applied per hectare (unit: t/ha), DWR–domestic waste water treatment rate, IWR–compliance rate of industrial waste water discharge, Fac–number of factories. The first half values of CFP, PP, DWR, and IWR refer to the average values of the indicators in 2005–2010 and the second half values of CFP, PP, DWR, and IWR refer to the average values of the indicators in 2011–2015. The first half values of Fac refer to the number of factories in 2010 and the second half values of Fac refer to the number of factories in 2011 since there was a sharp decrease in the number of factories in 2011 in Xuzhou.

## Supplementary Materials

The following are available online at https://www.mdpi.com/1660-4601/17/17/6388/s1, Table S1: The standard values for each class of the five main water quality parameters according to China’s Environmental Quality Standard for Surface Water (GB3838-2002). Table S2: The results of CWQII in Xuzhou section and comparison with other studies. Figure S1: Land use maps with 30 m resolution of Xuzhou City in 2005 and 2015. Figure S2: The results of the sequential Mann–Kendall test for the water quality time series in each site of Xuzhou section.
