# Supplementary material for: Are the Water Quality Improvement Measures of China’s South-to-North Water Diversion Project Effective? A Case Study of Xuzhou Section in the East Route
Source: Int J Environ Res Public Health. 2020 Sep 2;17(17):6388. doi: 10.3390/ijerph17176388 (PMC7503950; doi:10.3390/ijerph17176388)
Supplement: Supplementary file 1 [file ijerph-17-06388-s001.pdf]

International Journal of
Environmental Research and Public Health

Supplementary materials

Table S1. The standard values for each class of the five main water quality parameters according to China's Environmental Quality Standard for Surface Water (GB3838-2002).

Classes	DO	COD_{Mn}	BOD_5	NH_3-N	TP
**I**	7.5	2	3	0.15	0.02
**II**	6	4	3	0.5	0.1
**III**	5	6	4	1.0	0.2
**IV**	3	10	6	1.5	0.3
**V**	2	15	10	2.0	0.4

Table S2. The results of CWQII in Xuzhou section and comparison with other studies.

Location		Time	Indicators	Number of samples	CWQII (Avg±SD)	Reference
Honghu Lake	Hubei Province	2001-2011	DO, Chl-a, COD_{Mn}, NH_3-N, TN, TP	594	3.05±0.46	[1]
Donghu Lake	Hubei Province	2010	DO, COD_{Mn}, NH_3-N, TN, TP	96	3.67±0.28	[2]
Hua County	Gansu Province	2012	COD_{Mn}, COD_{Cr}, TN, TP, F^-	45	4.59±1.75	[3]
Chaohu Lake basin	Anhui Province	2015-2017	DO, COD_{Mn}, BOD_5, NH_3-N, TN, TP	72	3.60±1.00	[4]
Hanjiang River	Hubei Province	2017-2018	COD_{Mn}, BOD_5, TN, TP. NH_3-N, NO_3-N, NO_2-N, Chl-a, TOC	114	2.88±0.44	[5]
Xuzhou Section of the SNWD project	Jiangsu Province	2005-2010	DO, COD_{Mn}, BOD_5, NH_3-N, TP	432	2.79±0.53	This study
		2011-2015		360	2.61±0.33	

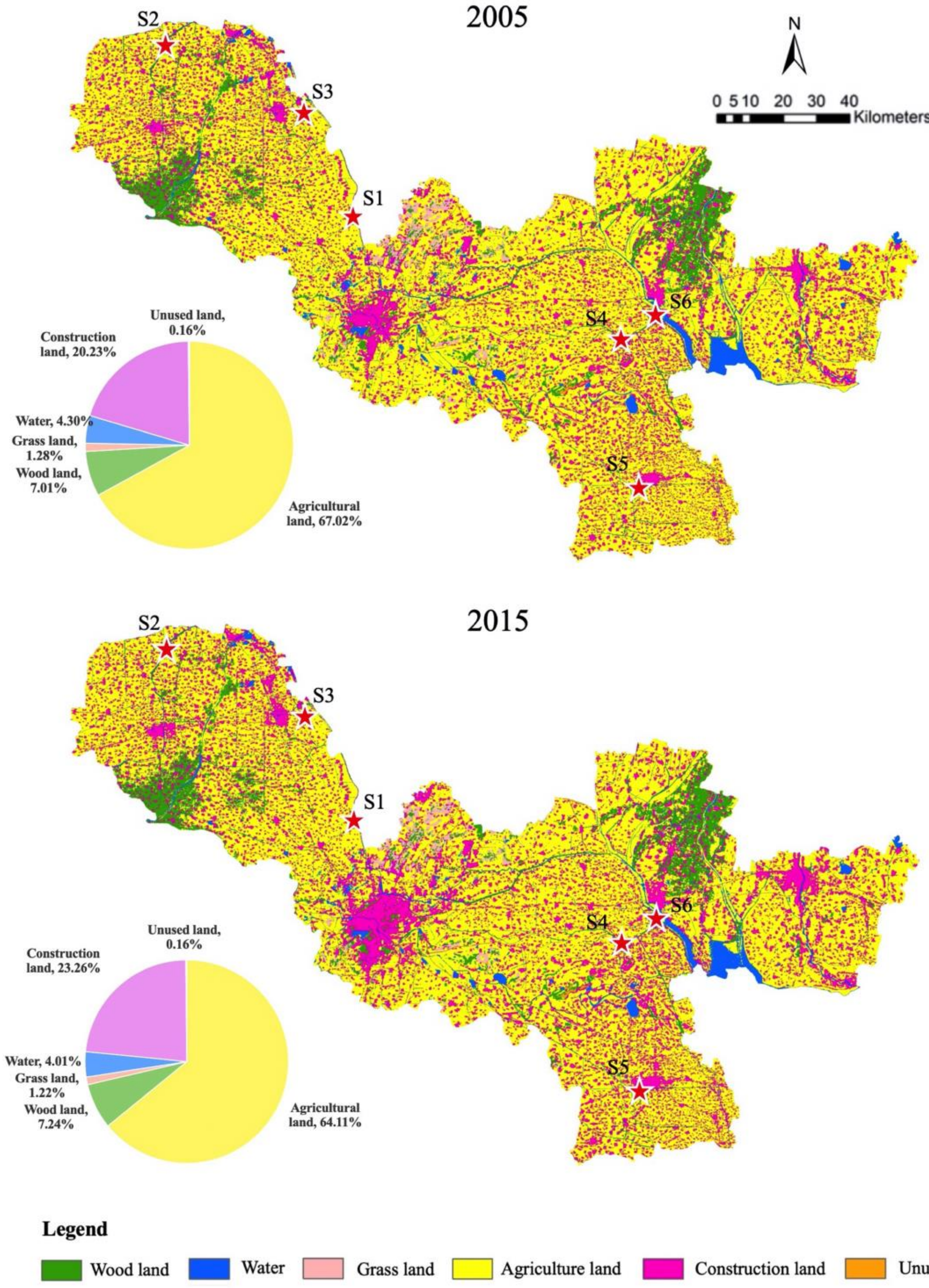

Figure S1. Land use maps with 30m resolution of Xuzhou City in 2005 and 2015. The land use data was derived from National Earth System Science Data Sharing Infrastructure of China (http://www.geodata.cn) and the Environment Data Cloud Platform (http://www.resdc.cn/data.aspx?DATAID=184, http://www.resdc.cn/data.aspx?DATAID=98).

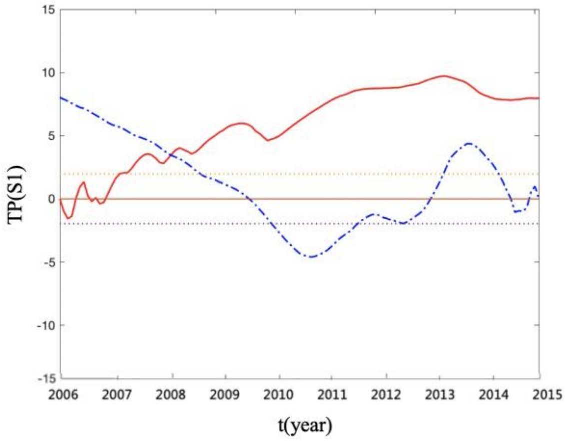
TP(S1)
15
10
5
0
-5
-10
-15
2006 2007 2008 2009 2010 2011 2012 2013 2014 2015
t(year)

(a) S1

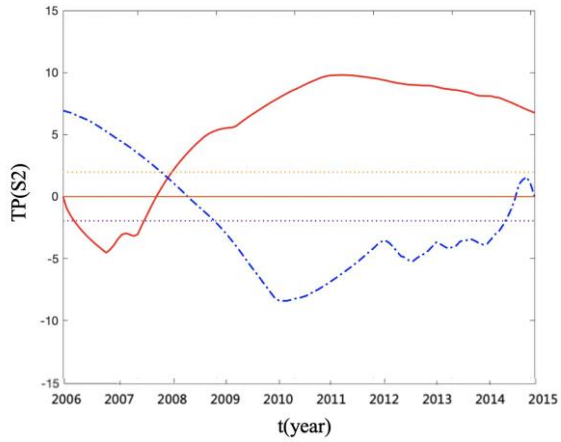
TP(S2)
15
10
5
0
-5
-10
-15
2006 2007 2008 2009 2010 2011 2012 2013 2014 2015
t(year)

(b) S2

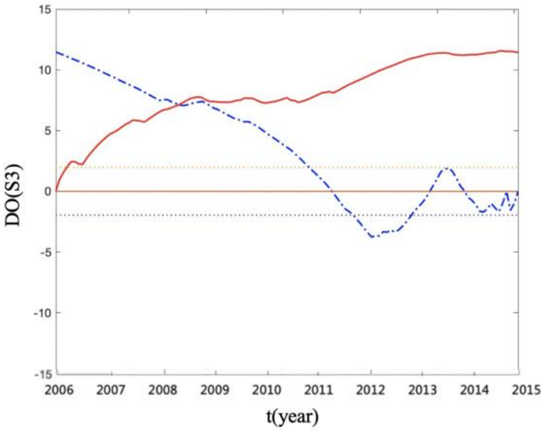
DO(S3)
15
10
5
0
-5
-10
-15
2006 2007 2008 2009 2010 2011 2012 2013 2014 2015
t(year)

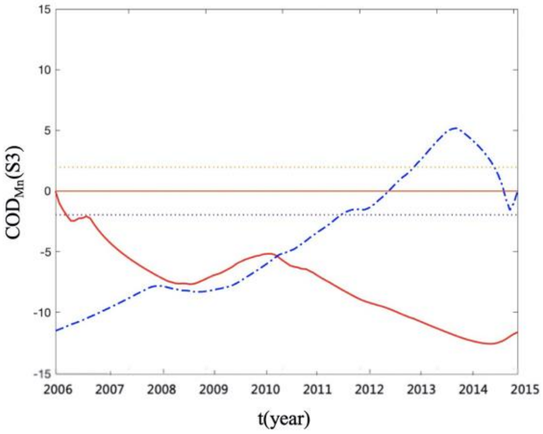
COD_{Mn}(S3)
15
10
5
0
-5
-10
-15
2006 2007 2008 2009 2010 2011 2012 2013 2014 2015
t(year)

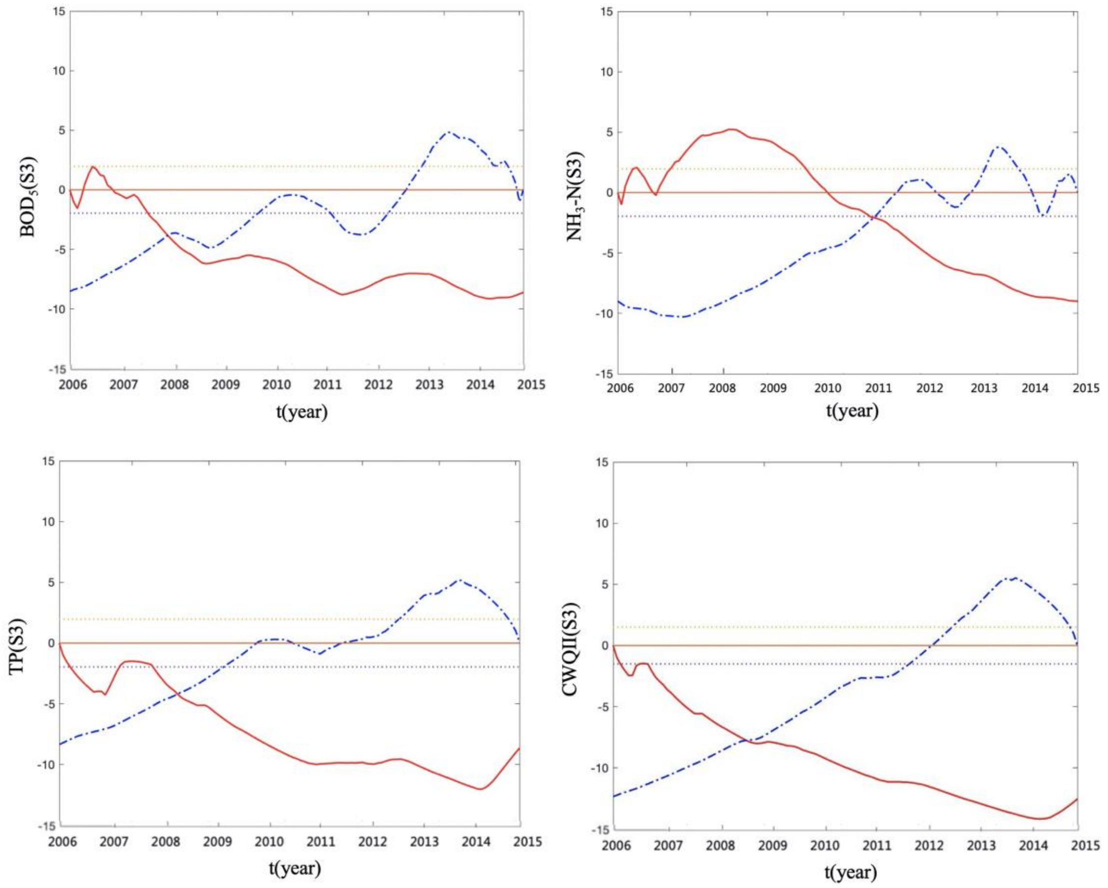
BOD5(S3)
NH3-N(S3)
TP(S3)
CWQII(S3)
t(year)
2006 2007 2008 2009 2010 2011 2012 2013 2014 2015
15 10 5 0 -5 -10 -15

(c) S3

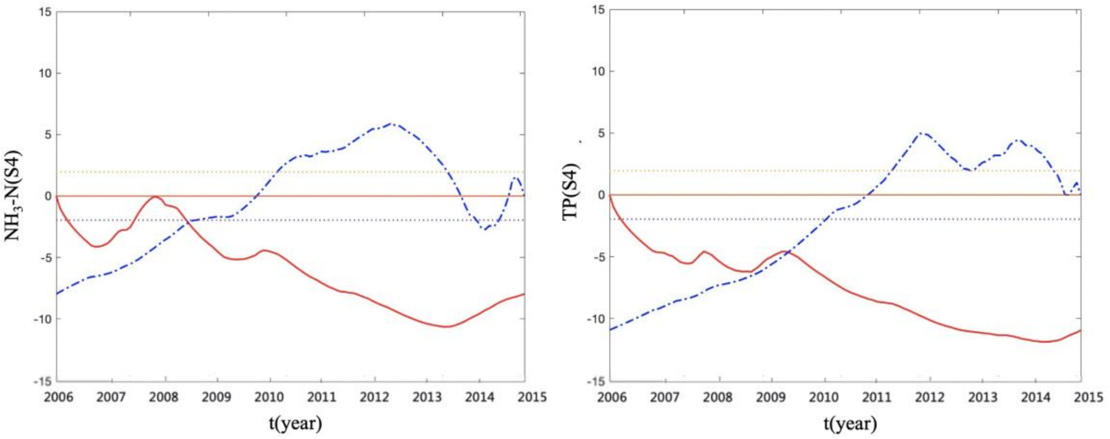
NH3-N(S4)
TP(S4)
t(year)
2006 2007 2008 2009 2010 2011 2012 2013 2014 2015
15 10 5 0 -5 -10 -15

(d) S4

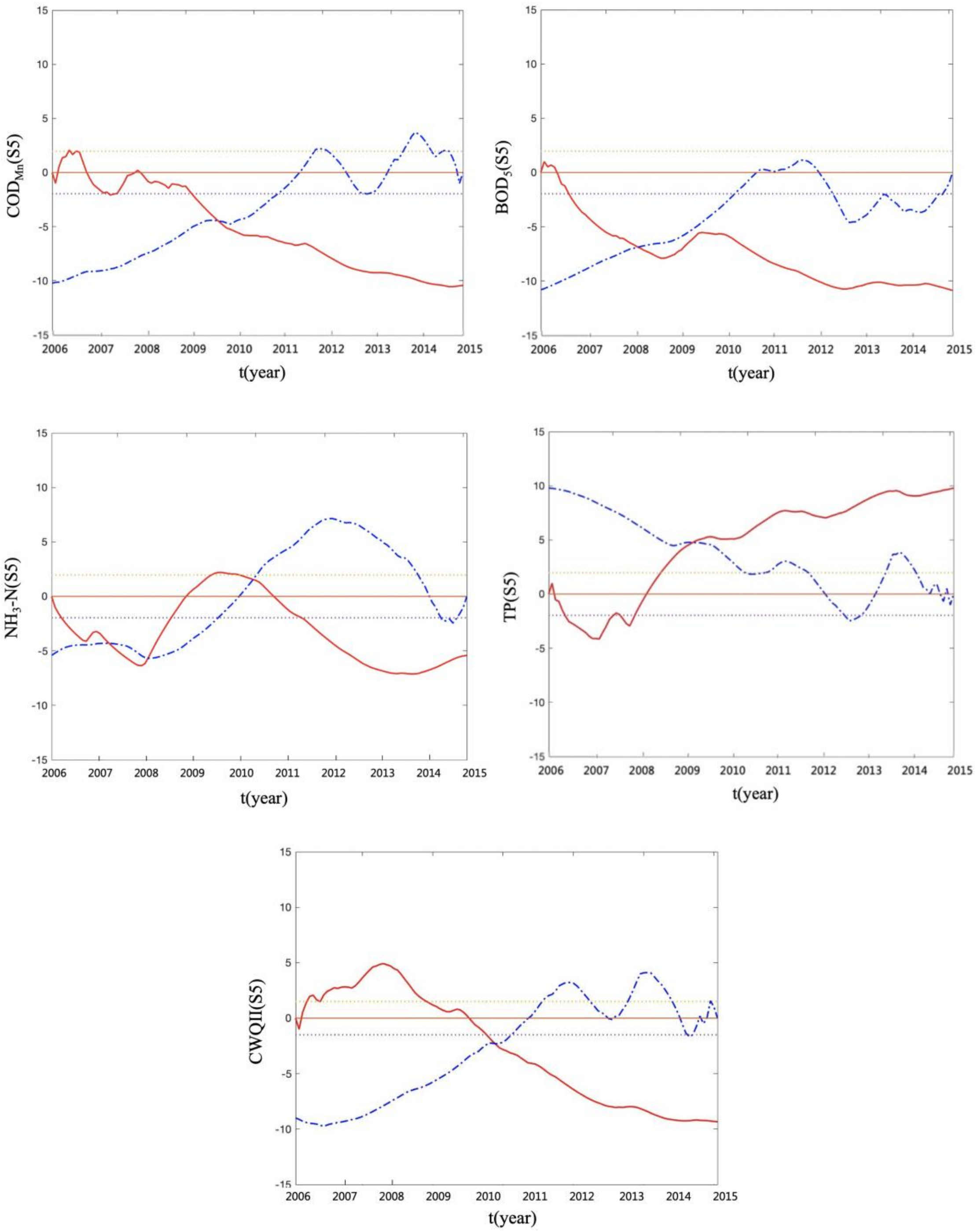

COD$_{Mn}$(S5)
BOD$_5$(S5)
NH$_3$-N(S5)
TP(S5)
CWQII(S5)
t(year)
15
10
5
0
-5
-10
-15
2006 2007 2008 2009 2010 2011 2012 2013 2014 2015

(e) S5

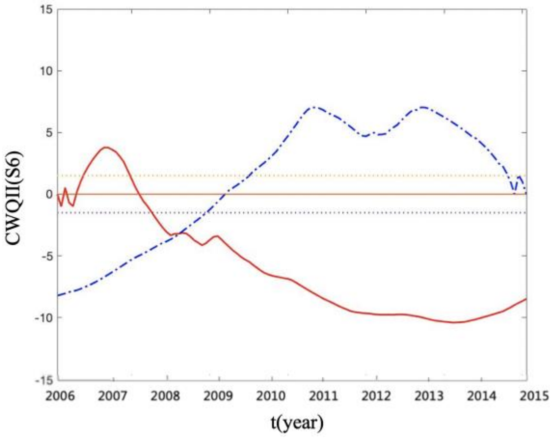

(f) S6

Figure S2. The results of the sequential Mann-Kendall test for the water quality time series in each site of Xuzhou section.
